# Cervical cancer screening practices among women in Ghana: evidence from wave 2 of the WHO study on global AGEing and adult health

**DOI:** 10.1186/s12905-020-00915-9

**Published:** 2020-03-05

**Authors:** Benedict N. L. Calys-Tagoe, Justice M. K. Aheto, George Mensah, Richard B. Biritwum, Alfred E. Yawson

**Affiliations:** 1 Department of Community Health, School of Public Health,College of Health Sciences, University of Ghana, Accra, Ghana; 2 Department of Biostatistics, School of Public Health, College of Health Sciences, University of Ghana, Accra, Ghana

**Keywords:** Cervical cancer, Pap smear test, Screening, Ghana, Sub-Saharan Africa, W.H.O. SAGE study

## Abstract

**Background:**

Cervical cancer is the third most common cancer that affects women worldwide. It has been and remains the leading cause of cancer mortality among women in Ghana. Despite the fact that cervical cancer is preventable through early detection and treatment of precancerous lesions, anecdotal evidence from gynaecological clinics in Ghana indicates that most patients present with a late stage of the disease. This study assesses the cervical cancer screening practices among women in Ghana.

**Methods:**

Data from the World Health Organization’s (WHO) multi-country Study on AGEing and adult health (SAGE) wave 2 conducted between 2014 and 2015 in Ghana was used. We employed binary logistic regression models to analyse data on 2711 women to examine factors associated with having pelvic examination among women aged ≥18 years. Among those who had pelvic examination, we applied binary logistic regression models to analyse factors associated with receiving Pap smear test as a subgroup analysis.

**Results:**

Of the 2711 women aged 18 years or older surveyed, 225 (8.3%) had ever had a pelvic examination and only 66 (2.4%) of them reported ever having done a Pap smear test. For those who had pelvic examination, only 26.94% had Pap smear test. Ethnic group, marital status, father’s educational level and difficulty with self-care were independently associated with undergoing pelvic examination. Only age and healthcare involvement were independently associated with pelvic examination within the past 5 years to the survey. Marital status, satisfaction with healthcare and healthcare involvement were independently associated with Pap smear test.

**Conclusion:**

Even though cervical cancer is preventable through early detection of precancerous lesions using Pap smear test, the patronage of this screening test is still very low in Ghana. Factors influencing the low patronage in Ghana include the marital status of women, their level of satisfaction with healthcare as well as their level of involvement with healthcare. These may be the consequences of a weak health system and the lack of a national policy on cervical cancer screening.

## Background

Cervical cancer is the third most common cancer that affects women worldwide and kills nearly 300,000 women annually [28]. The disease disproportionately affects poor people with at least 80% of cervical cancer deaths occurring in developing countries [19]. While cervical cancer is gradually becoming a rare disease in many developed countries, the same cannot be said of its occurrence in developing countries [11, 25]. It has been and remains the leading cause of cancer death among women in Ghana [29, 30].

Cervical cancer is however, one of the preventable human cancers; its prevention is based on the early diagnosis and treatment of precancerous lesions [8]. The screening test involves a pelvic examination during which a smear of cervical cells is taken for the Papanicolau test (Pap smear test). Visual inspection with acetic acid (VIA) could also be done during the pelvic examination. Anecdotal evidence from gynaecological clinics in Ghana indicates that most patients present with a late stage of the disease in spite of the fact that it is preventable. The major reason for the late presentation is the absence or very low coverage for cervical cancer screening in several African countries, [7, 13], including Ghana. Several reasons have been proffered for the low coverage of cervical cancer screening services in less developed countries. These include; the ignorance about the disease and its screening practices as well as perceptions and attitudes based on cultural and religious beliefs [9, 22]. The poor health infrastructure and other competing health interests may be other reasons for this low coverage [5, 6, 14].

To the best of our knowledge, there is no published data on the uptake of cervical cancer screening using a nationally representative sample in Ghana. In this study therefore, we aimed at determining the uptake of cervical cancer screening (pelvic examination +/− Pap smear test) and the factors influencing this uptake.

## Methods

We used data from the World Health Organization’s (WHO) study on global AGEing and adult health in Ghana which is part of a nationwide representative and a multi-country longitudinal study conducted among six countries. The study collects data to augment available sources of ageing data for informed health policy and programmes. The World Health Organization and the University of Ghana Medical School through the Department of Community Health collaborated to implement the Wave 2 of the study between 2014 and 2015. The methods used in the survey is available elsewhere [12].

### Study population

The study comprised of participants aged ≥50 years and additional smaller sample of participants between the ages of 18–49 years old were interviewed. Some of the data collected include subjective wellbeing and quality of life, health care utilization and preventive health behaviours, risk factors and perceived health status, socio-demographic and work history, chronic health conditions and health services coverage among others. This study was based on all ages of women in the study. Additional details about the study can be found at the WHO website (http://www.who.int/healthinfo/sage/cohorts/en/).

### Outcome variable

The WHO SAGE asked two specific questions: (1) ‘When was the last time you had a pelvic examination, if ever?’ (and provided a follow specific explanation as ‘By pelvic examination, I mean when a doctor or nurse examined your vagina and uterus?’) and (2) ‘The last time you had the pelvic examination, did you have a PAP smear test?’ (and provided a follow specific explanation as ‘By PAP smear test, I mean did a doctor or nurse use a swab or stick to wipe from inside your vagina, take a sample and send it to a laboratory?’). Thus, the main outcome variables in this study are (i) whether a woman ever received a pelvic examination (as a screening tool for cervical cancer) and (ii) whether a woman ever had a pap smear test. These are self-reports based on the questions, “When was the last time you had a pelvic examination, if ever?” and “The last time you had the pelvic examination, did you have a PAP smear test?” The responses were then categorized as Never (coded as 0) or ever had pelvic examination (coded as 1), and No (coded as 0) and Yes (coded as 1) for pap smear test. A secondary outcome of interest is whether a woman had pelvic examination within the last 5 years or not. Women who had pelvic examination within the last 5 years were categorized as Yes (coded as 1) and those who had it more than 5 years ago were categorized as No (coded as 0).

### Explanatory variables

The study considered factors such as age, ethnicity, marital status, father’s education, religion, health state report, healthcare involvement and difficulty with self-care. The literature on factors influencing health outcomes and healthcare seeking behaviours, especially in developing countries informed the inclusion of these variables [1, 10, 17, 21, 23].

### Statistical analysis

Binary logistic regression analyses were employed to identify factors associated with pelvic examination and Pap smear test. Analysis was conducted on 2711 study participants with complete measurements on pelvic examination status and potential risk factors considered in the study. A further subgroup analysis was conducted on those who ever had pelvic examination only. The group was categorised into those who had the examination within ≤5 years and those who had it > 5 years ago. Finally, a binary logistic regression model was applied on 242 individuals with complete measurements on Pap smear test while adjusting for potential risk factors.

We applied the Hosmer-Lemeshow [18] test to establish the fits of the multivariable models. To examine multicollinearity, the variance inflation factor (VIF) was applied. VIF value < 10 was considered acceptable [16]. STATA Version 14 [26] was used for data analyses. A *p*-value< 0.20 on a bivariate logistic regression was used to select candidate set of covariates for multivariable logistic regression. We declare statistical significance at *P*-value < 0.05.

### Ethical requirements

The SAGE survey was approved by the World Health Organization’s Ethical Review Board (reference number RPC149) and the University of Ghana College of Health Sciences, Ethical and Protocol Review Committee. Written informed consent was obtained from all study respondents.

## Results

A total of 225 of the women ever had pelvic examination out of which 66 of them had pap smear test. Seventy-four (74) of those in the age group 50–59 ever had pelvic examination and 14 of those aged 50–59 and 60–69 ever had pap smear test while 95 of those who were currently married ever had pelvic examination and 22 of them had pap smear test. Among women who rated their health state today as good/very good, 159 of them ever had pelvic examination while 42 of them had pap smear test. For women who rated themselves as having no difficulty with self-care, 200 of them ever had pelvic examination while 55 of them had pap smear test (Table 1).

**Table 1:** Background characteristics of women by pelvic examination and pap smear test status

Characteristics	Pelvic examination status		Had pap smear test	
	Never	Less than a year or more	No	Yes
**Age of respondent (years)**				
18–29	190	13	8	10
30–39	185	30	24	10
40–49	215	31	25	7
50–59	745	74	63	14
60–69	551	47	36	14
70 or more	607	30	23	11
**Marital status**				
Never married	210	11	2	10
Currently married	1079	95	82	22
Cohabiting	39	4	3	2
Separated/divorced	324	43	30	15
Widowed	841	72	62	17
**Father’s education**				
No formal education	1843	143	120	38
Primary or less	306	20	17	5
Secondary or higher	344	62	42	23
**Ethnic group**				
Akan	1297	114	88	38
Ewe	136	31	24	7
Ga-Adangbe	335	24	23	6
Guan	81	14	13	2
Northern dialect	644	42	31	13
**Religion**			–	
No religion	58	1		
Christianity	1930	184		
Islam	407	36		
African traditional	84	3		
Others^a^	14	1		
**Health state today**				
Good/very good	1624	159	131	42
Moderate	646	53	38	19
Bad/very bad	218	13	10	5
**Satisfaction with health care**	–			
Satisfied/very satisfied			158	53
Indifferent			16	9
Dissatisfied/very dissatisfied			3	4
**Rate health care involvement**	–			
Good/very good			44	44
Moderate			28	9
Bad/very bad			106	13
**Difficulty with self-care**				
None	1964	200	164	55
Mild	378	17	11	7
Moderate	114	6	3	3
Severe/extreme	36	1	0	1

^a^Buddhism, Chinese traditional religion, Hinduism.

### Factors associated with having pelvic examination

Factors associated with having pelvic examination in the univariable binary logistic regression include age, ethnic group, marital status, father’s educational level and difficulty with self-care. In the multivariable binary logistic regression model, ethnic group, marital status, father’s educational level and difficulty with self-care were independently associated with undergoing pelvic examination. Those from the Ewe ethnic group had increased odds (OR = 2.65, 95% CI: 1.68, 4.17) of undergoing pelvic examination compared to their counterpart from the Akan ethnic group. Women who were separated/divorced (OR = 3.57, 95% CI: 1.64, 7.75) and widowed (OR = 3.43, 95% CI: 1.58, 7.43) had higher chance of undergoing pelvic examination compared to women who were single and women who had fathers with secondary education or higher had increased odds (OR = 1.91, 95% CI: 1.34, 2.72) of undergoing pelvic examination compared to women who had fathers with no formal education. Those who rated their difficulty with self-care as mild had decreased odds (OR = 0.57, 95% CI: 0.34, 0.97) of undergoing pelvic examination compared to those who rated themselves as having no such difficulty (Table 2).

**Table 2:** Factors associated with pelvic examination status from binary logistic regression (*n* = 2711)

Characteristics	Unadjusted model		Adjusted model	
	UOR (95% CI)	***P***-value	AOR (95% CI)	***P***-value
**Age of respondent (years)**				
18–29	ref		ref	
30–39	2.37 (1.2, 4.69)	0.013	1.79 (0.85, 3.77)	0.122
40–49	2.11 (1.07, 4.14)	0.031	1.35 (0.63, 2.87)	0.441
50–59	1.45 (0.79, 2.67)	0.231	0.98 (0.48, 1.98)	0.95
60–69	1.25 (0.66, 2.35)	0.497	0.75 (0.35, 1.59)	0.455
70 or more	0.72 (0.37, 1.41)	0.342	0.46 (0.21, 1.04)	0.064
**Ethnic group**				
Akan	ref		ref	
Ewe	2.59 (1.68, 4)	< 0.001	2.65 (1.68, 4.17)	< 0.001
Ga-Adangbe	0.82 (0.52, 1.29)	0.38	0.88 (0.55, 1.4)	0.598
Guan	1.97 (1.08, 3.58)	0.027	2.18 (1.15, 4.13)	0.017
Northern dialect	0.74 (0.51, 1.07)	0.11	0.87 (0.59, 1.28)	0.468
**Marital status**				
Never married	ref		ref	
Currently married	1.68 (0.88, 3.19)	0.113	2.22 (1.07, 4.6)	0.031
Cohabiting	1.96 (0.59, 6.46)	0.27	1.7 (0.49, 5.92)	0.405
Separated/divorced	2.53 (1.28, 5.02)	0.008	3.57 (1.64, 7.75)	0.001
Widowed	1.63 (0.85, 3.14)	0.14	3.43 (1.58, 7.43)	0.002
**Father’s educational level**				
No formal education	ref		ref	
Primary or less	0.84 (0.52, 1.37)	0.487	0.72 (0.43, 1.19)	0.195
Secondary or higher	2.32 (1.69, 3.2)	< 0.001	1.91 (1.34, 2.72)	< 0.001
**Religion**				
No religion	ref			
Christian	5.53 (0.76, 40.15)	0.091		
Islam	5.13 (0.69, 38.13)	0.11		
African traditional	2.07 (0.21, 20.41)	0.533		
Others^a^	4.14 (0.24, 70.38)	0.325		
**Rate your health today**				
Good/very good	ref		ref	
Moderate	0.84 (0.61, 1.16)	0.285	1.01 (0.71, 1.44)	0.947
Bad/very bad	0.61 (0.34, 1.09)	0.095	0.84 (0.45, 1.59)	0.599
**Difficulty with self-care**				
None	ref		ref	
Mild	0.44 (0.27, 0.73)	0.002	0.57 (0.34, 0.97)	0.037
Moderate	0.52 (0.22, 1.19)	0.121	0.71 (0.3, 1.7)	0.441
Severe/extreme	0.27 (0.04, 2)	0.201	0.36 (0.05, 2.8)	0.328

*UOR* Unadjusted odds ratio, *AOR* Adjusted odds ratio, *CI* Confidence interval, *ref*. Reference category, ^a^(e.g. Buddhism, Chinese traditional religion, Hinduism).

Factors associated with pelvic examination within the past 5 years to the survey in the univariable binary logistic regression include age, ethnic group, marital status, father’s educational level, health state today and healthcare involvement. In the multivariable binary logistic regression model, only age and healthcare involvement were independently associated with pelvic examination within the past 5 years to the survey. Women in the age group 50–59 (OR = 0.03, 95% CI: 0.00, 0.25), 60–69 (OR = 0.03, 95% CI: 0.00, 0.33) and ≥ 70 years (OR = 0.08, 95% CI: 0.01, 0.89) had decreased odds of receiving pelvic examination within the past 5 years to the survey compared to those in age group 18–29 years. Those who rated their health care involvement as bad/very bad had reduced odds (OR = 0.32, 95% CI: 0.12, 0.84) of receiving pelvic examination within the past 5 years to the survey compared to their counterparts who rated their health care involvement as good/very good (Table 3).

**Table 3:** Factors associated with pelvic examination within the past 5 years to the survey from binary logistic regression (*n* = 224)

Characteristics	Unadjusted model		Adjusted model	
	UOR (95% CI)	***P***-value	AOR (95% CI)	***P***-value
**Age of respondent (years)**				
18–29	ref		ref	
30–39	1.18 (0.19, 7.43)	0.859	2.46 (0.28, 21.74)	0.417
40–49	0.13 (0.02, 0.7)	0.017	0.22 (0.03, 1.96)	0.176
50–59	0.02 (0, 0.1)	< 0.001	0.03 (0, 0.25)	0.001
60–69	0.02 (0, 0.1)	< 0.001	0.03 (0, 0.33)	0.004
70 or more	0.03 (0, 0.18)	< 0.001	0.08 (0.01, 0.89)	0.04
**Ethnic group**				
Akan	ref			
Ewe	1.12 (0.45, 2.79)	0.807		
Ga-Adangbe	1.93 (0.76, 4.91)	0.166		
Guan	1.29 (0.37, 4.44)	0.688		
Northern dialect	2.19 (1.03, 4.65)	0.041*		
**Marital status**				
Never married	ref		ref	
Currently married	0.14 (0.03, 0.66)	0.014	1.25 (0.11, 14.16)	0.856
Cohabiting	0.67 (0.04, 10.25)	0.771	0.37 (0.02, 8.85)	0.543
Separated/divorced	0.06 (0.01, 0.32)	0.001	0.57 (0.04, 7.45)	0.67
Widowed	0.03 (0.01, 0.15)	< 0.001	0.36 (0.03, 4.77)	0.438
**Father’s educational level**				
No formal education	ref		ref	
Primary or less	2.62 (1, 6.86)	0.049	1.37 (0.36, 5.29)	0.644
Secondary or higher	1.76 (0.92, 3.37)	0.086	1.35 (0.51, 3.56)	0.54
**Rate your health today**				
Good/very good	ref		ref	
Moderate	0.41 (0.19, 0.9)	0.026	1.03 (0.36, 2.95)	0.955
Bad/very bad	0.6 (0.16, 2.27)	0.452	0.93 (0.12, 7.32)	0.943
**Rate health care involvement**				
Good/very good	ref		ref	
Moderate	0.47 (0.2, 1.14)	0.096	0.57 (0.17, 1.92)	0.361
Bad/very bad	0.39 (0.2, 0.73)	0.004	0.32 (0.12, 0.84)	0.021

*UOR* Unadjusted odds ratio, *AOR* Adjusted odds ratio, *CI* Confidence interval, *ref*. Reference category .

### Factors associated with pap smear test

Factors associated with Pap smear test in the univariable binary logistic regression include age, marital status and healthcare involvement. In the multivariable binary logistic regression model, marital status, satisfaction with healthcare and healthcare involvement were independently associated with Pap smear test. Those who are currently married (OR = 0.12, 95% CI: 0.02, 0.89), and widowed (OR = 0.07, 95% CI: 0.01, 0.62) had reduced odds of receiving pap smear test compared to women who are never married. Women who rated their satisfaction with health care as dissatisfied/very dissatisfied had higher odds (OR = 16.75, 95% CI: 2.85, 98.33) of receiving pap smear test compared to those who rated their satisfaction with health care as satisfied/very satisfied. Those who rated their health care involvement as moderate (OR = 0.2, 95% CI: 0.07, 0.58) and bad/very bad (OR = 0.09, 95% CI: 0.04, 0.21) had decreased odds of receiving pap smear test compared to the women who rated themselves as good/very good (Table 4).

**Table 4:** Factors associated with pap smear test status from binary logistic regression (*n* = 242)

Characteristics	Unadjusted model		Adjusted model	
	UOR (95% CI)	***P***-value	AOR (95% CI)	***P***-value
**Age of respondent (years)**				
18–29	ref		ref	
30–39	0.33 (0.1, 1.09)	0.07	0.56 (0.11, 3.01)	0.5
40–49	0.22 (0.06, 0.78)	0.019	0.64 (0.11, 3.91)	0.63
50–59	0.18 (0.06, 0.53)	0.002	0.45 (0.09, 2.35)	0.35
60–69	0.31 (0.1, 0.95)	0.04	0.88 (0.16, 5.03)	0.89
70 or more	0.38 (0.12, 1.24)	0.109	1.48 (0.22, 9.95)	0.69
**Ethnic group**		0.56		
Akan	ref			
Ewe	0.68 (0.27, 1.7)	0.405		
Ga-Adangbe	0.6 (0.23, 1.6)	0.311		
Guan	0.36 (0.08, 1.66)	0.188		
Northern dialect	0.97 (0.46, 2.06)	0.939		
**Marital status**				
Never married	ref		ref	
Currently married	0.05 (0.01, 0.26)	< 0.001	0.12 (0.02, 0.89)	0.04
Cohabiting	0.13 (0.01, 1.39)	0.092	0.31 (0.02, 5.09)	0.41
Separated/divorced	0.1 (0.02, 0.52)	0.006	0.17 (0.02, 1.41)	0.1
Widowed	0.05 (0.01, 0.27)	< 0.001	0.07 (0.01, 0.62)	0.02
**Father’s educational level**				
No formal education	ref		ref	
Primary or less	0.93 (0.32, 2.69)	0.892	0.4 (0.13, 1.28)	0.12
Secondary or higher	1.73 (0.92, 3.23)	0.086	1.18 (0.51, 2.7)	0.7
**Rate your health today**				
Good/very good	ref			
Moderate	1.56 (0.81, 2.99)	0.181		
Bad/very bad	1.56 (0.5, 4.82)	0.44		
**Satisfaction with health care**				
Satisfied/very satisfied	ref		ref	
Indifferent	1.68 (0.7, 4.02)	0.246	2.91 (0.91, 9.33)	0.07
Dissatisfied/very dissatisfied	3.97 (0.86, 18.34)	0.077	16.75 (2.85, 98.33)	< 0.001
**Rate health care involvement**				
Good/very good	ref		ref	
Moderate	0.32 (0.14, 0.76)	0.01	0.2 (0.07, 0.58)	< 0.001
Bad/very bad	0.12 (0.06, 0.25)	< 0.001	0.09 (0.04, 0.21)	< 0.001
**Difficulty with self-care**				
None	ref			
Mild	1.9 (0.7, 5.14)	0.207		
Moderate	2.98 (0.58, 15.21)	0.189		

*UOR* Unadjusted odds ratio, *AOR* Adjusted odds ratio, *CI* Confidence interval, *ref*. Reference category.

## Discussion

In this analysis, 8.3% of women in Ghana have ever had a pelvic examination compared to the 12% prevalence in wave 1 of the same survey. The study further revealed that only 2.4% of women in Ghana have ever had a Pap smear compared to a prevalence of 3.1% in wave 1. It is possible that some of the women who had pelvic examination done could have been screened for cervical cancer using the visual inspection of the cervix with acetic acid (VIA) in which case the proportion of women screened for cervical cancer will have been greater than 2.4%. The WHO SAGE study, however, did not inquire about VIA. Most women will have a pelvic examination done for other gynaecological reasons other than screening for cervical cancer. This explains the nearly three-fold difference in the proportions of those who have had a pelvic examination and those who had a Pap smear test done. The low patronage for cervical cancer screening using Pap smear has been reported in previous publications in Ghana [2–4, 15]. This low patronage for cervical cancer screening is not peculiar to Ghana but has been reported in other developing countries [8, 20, 24]. In India, the country with the largest burden of cervical cancer worldwide, patronage was only 11.6% among nurses (health workers who are expected to know and have the right attitude towards screening for cervical cancer) [27]. This is in sharp contrast to what pertains in developed countries like the United States where over 80% of women sampled had had a Pap smear test in the preceding 3 years (www.progressreport.cancer.gov/detection/cervical_cancer).

Some of the reasons attributed to the low patronage in those studies included the lack of a national cervical cancer screening programme, the effect of education [4] and the lack of financial resources [8]. The rate in health care involvement as well as level of satisfaction/dissatisfaction with health care can both be causes as well as consequences of having had a pelvic examination (and by extension Pap smear test) and these should not be overlooked in finding answers to the low patronage being reported.

There is still no national policy or programme regarding cervical cancer screening in Ghana and that could be contributing to the persisting low patronage of cervical cancer screening in Ghana.

In this analysis, however, factors that were found to be associated with patronage of Pap smear were; marital status, level of satisfaction with healthcare and level of involvement with healthcare. Marital status was found to be a predictor for having a Pap smear test. Women who were currently married (*p* = 0.04) and those who had lost their spouses (*p* = 0.02) were found to be less likely to undergo screening for cervical cancer. People in stable marital relationships or those who have lost their spouses may not consider themselves to be at risk of cervical cancer since they are less likely to have multiple sexual partners or be sexually active, all of which increase one’s risk of acquiring cervical cancer.

Surprisingly, women who were dissatisfied with the healthcare they received upon previous contact with the health care delivery system were found to have significantly higher odds of undergoing Pap smear compared to those who were satisfied with the health care they received. Being dissatisfied with healthcare service received could make an individual seek a second or even a third opinion and be willing to be subjected to varied screening/diagnostic tests. This may be a possible reason for their increased odds.

Not being very concerned about one’s healthcare (those who rated their involvement in healthcare as moderate or bad) was found to significantly decrease the odds (*p* < 0.001) of undergoing a Pap smear test. This observation came as no surprise as people who are very concerned about their health and healthcare will stop at nothing (including screening for cervical cancer, among others) to convince themselves of their health status at any point in time.

## Conclusion

Even though cervical cancer is preventable through early detection of precancerous lesions using Pap smear test, the patronage of this screening test is still very low in Ghana. Factors influencing the low patronage in Ghana include the lack of a national policy on cervical cancer screening, the marital status of women, their level of satisfaction with healthcare as well as their level of involvement with healthcare. Developing and implementing a clear national policy on cervical cancer screening coupled with a structured national health educational intervention programme by the Ministry of Health/Ghana Health Service is worth considering. This could, improve knowledge and reduce the missed opportunities associated with cervical cancer screening in Ghana.

Secondly, an improvement in the overall healthcare delivery system will encourage more women to patronize these services as more of them are likely to be satisfied with improved services. Women should also be empowered and encouraged to take responsibility for their health in general and cervical cancer screening in particular.

## Data Availability

Data and materials freely available upon making official request to WHO-SAGE Team through the WHO website at http://www.who.int/healthinfo/sage/cohorts/en/.

## Abbreviations

SAGE: Study on AGEing and adult health
VIA: Visual inspection with acetic acid
VIF: Variance inflation factor
WHO: World Health Organization
